# Visual categorisation of the Arch Index: a simplified measure of foot posture in older people

**DOI:** 10.1186/1757-1146-5-S1-P15

**Published:** 2012-04-10

**Authors:** Hylton B Menz, Mohammad R Fotoohabadi, Elin Wee, Martin J Spink

**Affiliations:** 1 Musculoskeletal Research Centre, La Trobe University, Bundoora, Victoria 3086, Australia

## Background

Many foot posture measurement approaches are not suitable for routine use as they are time-consuming or require specialised equipment and/or clinical expertise. The objective of this study was to develop and evaluate a simple visual assessment tool for foot posture assessment based on the Arch Index (AI) [1].

## Materials and methods

**Figure 1:**
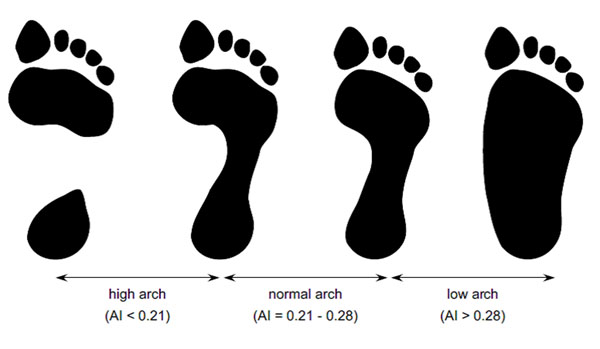
The simplified AI visual assessment tool.

Fully weightbearing footprints from 602 people aged 62 to 96 years were obtained using a carbon paper imprint material, and cut-off AI scores dividing participants into three categories (high, normal and low) were determined. A visual tool was created using representative examples for the boundaries of each category (Figure 1). Two examiners used the tool to independently grade the footprints of 60 participants (20 for each of the three categories, randomly presented), and then repeat the process two weeks later. Inter- and intra-tester reliability were determined and the validity of the examiner’s assessments was evaluated by comparing their categorisations to the actual AI score.

## Results

Inter- and intra-tester reliability of the examiners was almost perfect (percentage agreement = 93 to 97%; Spearman’s rho = 0.91 to 0.95, and weighted kappas = 0.85 to 0.93). Examiner’s scores were strongly correlated with actual AI values (Spearman’s rho = 0.91 to 0.94 and significant differences between all categories with ANOVA; p<0.001) and AI categories (percentage agreement = 95 to 98%; Spearman’s rho = 0.89 to 0.94, and weighted kappas = 0.87 to 0.94). There was a slight tendency for examiners to categorise participants as having higher arches than their AI scores indicated.

## Conclusions

Foot posture can be quickly and reliably categorised as high, normal or low in older people using a simplified visual categorisation tool based on the AI.
